# 

**Published:** 1907-01-26

**Authors:** 


### BOOKS RECEIVED.

J. B. LIPPINCOTT CO.

"Pediatrics: The Hygienic and Medical Treatment of Children." By T. M. Rotch, M.D.

SCOTT AND BOWNE, LTD.

"Doctors' and Nurses' Diary and Emergency Note-book," 1907.

THE prize of £50 given annually by Sir John Craggs, M.V.O., to the member of the London School of Tropical Medicine who makes the most valuable contribution to tropical medicine during the year, has just been awarded to Fleet-Surgeon Bassett-Smith.

### NOTICE TO CORRESPONDENTS.

All MSS., Letters, Books for Review, and other matters intended for the Editor should be addressed THE EDITOR, "THE HOSPITAL," THE HOSPITAL BUILDINGS, 28 & 29 SOUTHAMPTON STREET, STRAND, W.C.
The Editor cannot undertake to return rejected MS., even when accompanied by stamped directed envelope.

**Notice to Advertisers and Subscribers.**

All Advertisements, Orders for Copies of THE HOSPITAL, and Business Communications should be addressed to **THE MANAGER (Not to the Editor),** THE HOSPITAL, 28 & 29 Southampton Street, Strand London, W.C.
The Cost of Subscription, post free, is as follows:—Per week, 3½d.; per quarter, 3s. 3d.; per half-year, 6s. 6d.; per annum, 13s. Foreign and Colonial:—Per annum, 19s. 6d., payable, in all cases, in advance.

**NOTICE.—Vol. XL. of "The Hospital" (April to September 1906), in handsome cloth binding will shortly be on sale at the office of this Journal price 10s. 6d. Cases for binding the Numbers contained in this Volume 2s. Index to Vol. XL., 3½d., post free.**
**The Monthly Part for January is now ready. Price 1s., by post, 1s. 3d.**

### New Books for Nurses

A NEW AND IMPORTANT BOOK ON

### SKIN DISEASES:

THEIR NURSING AND PRACTICAL MANAGEMENT.

**2s. 6d. net.** 2s. 9d. post free.

By G. NORMAN MEACHEN, M.D., M.B., M.R.C.S., L.R.C.P.

**2s. 6d. net.** 2s. 9d. post free.

This is the only work on Skin Diseases written especially for Nurses. It deals with all the more common diseases and describes very fully usual forms of treatment. The work cannot fail to be of the utmost value to Nurses, and should prove a useful book for the household.

A MANUAL FOR NURSES ON

### ABDOMINAL SURGERY.

**2s. 6d. net.** 2s. 9d. post free.

By HAROLD BURROWS, M.B., F.R.C.S.

**2s. 6d. net.** 2s. 9d. post free.

*CONTENTS*: Symptoms in Abdominal Disease—Peritonitis—Hernia—Affections of the Stomach—Affections of the Small Intestine—Intestinal Obstruction—Intussusception—Appendicitis—Affections of the Liver and Gall-bladder—Diseases of the Uterus and Appendages—Preparation for Abdominal Section—After-troubles and Complications of Abdominal Operations—Feeding.

You have often been in need of a Book

That would aid you in emergency. That would help you to recollect some point you had forgotten or about which you had a doubt.

That would prove of assistance in difficulty.

That would convey information clearly and concisely on Diseases; their Symptoms and Nursing Treatment.

### THE NURSE'S "ENQUIRE WITHIN"

is a Pocket Encyclopædia of valuable nursing information alphabetically arranged, and

Fills a Want that has long been felt by Nurses everywhere. It is bound in limp cloth boards with rounded corners, and its size and weight will admit of its being carried conveniently in the apron pocket without injury thereto.

**2s. net; 2s. 3d. post free.**

THE

3d. net. By post, 4½d. **CASE BOOK** 3d. net. By post, 4½d.

FOR

### PRIVATE NURSING,

WITH RULINGS FOR

**Day and Night Nurses' Reports.**

Comprising Time, Nourishment, Stimulants, Medicines, Urine, Bowels, Summary Remarks, &c. &c.

**This Case Book has been described by some of the highest Medical Authorities as "the most concise Nurses' Report Book issued."**

**The Scientific Press, Ltd.,**

*Catalogue of Nursing Manuals sent post free on application.*

Publishers of Nursing Text-Books, Charts, and Case-Books of all Descriptions.

28 & 29 SOUTHAMPTON STREET, STRAND, LONDON, W.C.

